# Experimental study of the influence of mode excitation on mode instability in high power fiber amplifier

**DOI:** 10.1038/s41598-019-45787-8

**Published:** 2019-06-28

**Authors:** Qiuhui Chu, Rumao Tao, Chengyu Li, Honghuan Lin, Yuying Wang, Chao Guo, Jianjun Wang, Feng Jing, Chuanxiang Tang

**Affiliations:** 1 Accelerator Laboratory, Department of Engineering Physics, Tsinghua University, Beijing, 100084 China; 2 Research Center of Laser Fusion, China Academy of Engineering Physics, Mianyang, 621900 China

**Keywords:** Fibre lasers, Optical physics

## Abstract

Mode instability with different mode excitation has been investigated by off-splicing the fusion point in a 4 kW-level monolithic fiber laser system, which reveals that the fiber systems exciting more high order mode content exhibits lower beam quality but higher mode instability threshold. The static-to-dynamic mode degradation and dynamic-only mode degradation have also been observed in the same high power fiber amplifier by varying the mode excitation, which implicates that the mode excitation plays an important role in mode characteristics in high power fiber lasers. By employing a seed with near fundamental mode beam quality, only dynamic mode degradation-mode instability sets in with negligible static beam quality degradation. Then the fusion point in the seed laser is offset spliced to excite high order mode. As the output power of the main amplifier scales, the beam quality degrades with the beam profile being static, and then the dynamic mode instability sets in, the power threshold of which is higher than that with good beam quality seed. We consider that the static mode degradation is caused by the presence of incoherent supposition of fundamental and high order mode, which leads to that the measured dynamic mode instability threshold is higher.

## Introduction

Transverse mode instability (TMI), which results to that the output beam profiles become unstable, and exhibit temporal fluctuation in the range of a few kHz^1,2^, is currently a major limitation on the power scalability of large mode area single mode Yb-doped fiber amplifiers^3,4^. Due to the far-reaching impact of TMI, lots of works have been carried out to gain a deep insight into this phenomenon, and some effective strategies have been proposed which leads to that the output power of fiber lasers with near diffraction limited beam quality have already been scaled up to several kilowatts^5–13^. By summarizing the recent reported results of multi-kilowatt high power fiber lasers, it is interesting to note that the output power of fiber lasers with high beam quality (M^2^ < 1.3) are limited by TMI to below 4.3 kW^14–19^, while those with low beam quality (M^2^ > 1.3) have been scaled up to far beyond 4.3 kW without observation of dynamic beam profile fluctuation of TMI^20–23^. It is well known that the M^2^ beam quality is a widely used characterization of mode content, which means that the mode excitation may induce such difference in those fiber laser systems and it is urgent to study the influence of mode excitation of seed laser on TMI. However, to the best of our knowledge, no investigation on the mode excitation of seed laser on TMI has been carried out in multi-kilowatt monolithic fiber laser systems except for some anecdotal reports in^24,25^.

In the paper, a 4 kW-level fiber laser system has been set up, and a study of TMI threshold for different beam quality by varying the mode excitation of the injecting seed laser has been carried out, which indicates that the beam quality of the laser system have impact on the characteristics of the TMI. To the best of our knowledge, the static-to-dynamic mode coupling (first steady-state power transfering to high order mode (HOM) and then dynamic power coupling between fundamental mode (FM) and HOM) and dynamic-only mode coupling (only dynamic power coupling between FM and HOM) in a same single-pass high power fiber amplifier by varying the mode excitation have been observed simultaneously for the first time, which is different from the type of the static and dynamic mode coupling reported in a double-pass rod-type fiber amplifier^26^.

## Laser Configuration

**Figure 1:**
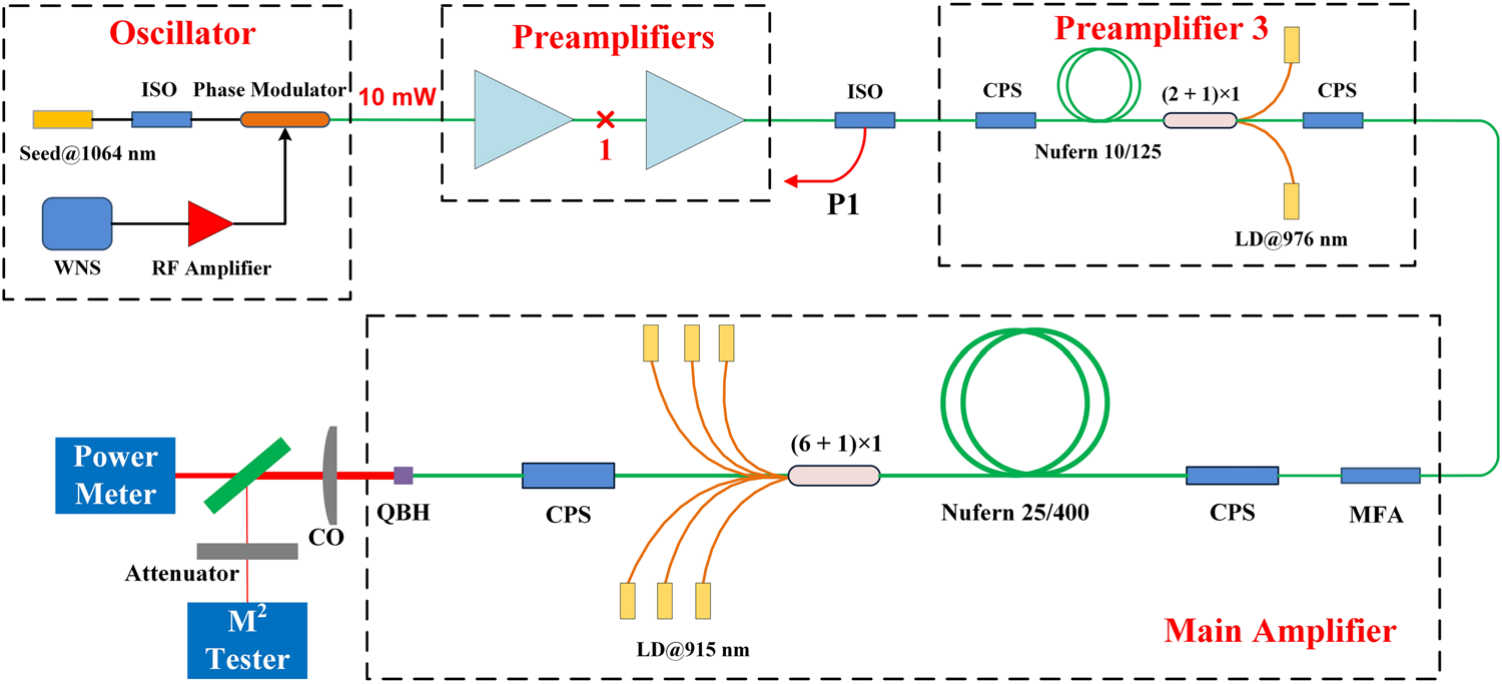
Experiment setup of the laser system.

Figure 1 shows the experiment setup of the narrow linewidth single pass fiber laser. The seed is a single frequency distributed feedback (DFB) laser with central wavelength being 1064 nm. To suppress the devastating SBS effect, the linewidth of the single frequency seed is broadened to 0.25 nm by phase modulation. Then, the output power of seed laser is amplified to 100 W by three stage preamplifiers. The active fibers of the three preamplifiers are Nufern 10/125 Yb-doped fiber with pump absorption factor of 4.1 dB/m at 975 nm. The isolators (ISOs) are applied to prevent damage from backward light, and the multi-mode fiber port of ISO is used to monitor backward power. The amplified seed laser is coupled into the main amplifier through a mode field adapter. To increase the TMI threshold, the counter pumping configuration has been employed in the main amplifier stage^10^. The main amplifier is made up of a counter (6 + 1) × 1 signal/pump combiner, a 18 m long Nufern 25/400 large mode area Yb-doped fiber with pump absorption factor of 0.6 dB/m at 915 nm, two cladding power strippers (CPSs) and an output quartz block holder (QBH). Wavelength-locked 915 nm laser diodes (LDs) are employed to pump the gain fiber and achieve higher TMI threshold^6^, and the CPSs are utilized to remove unwanted cladding light. To obtain good beam quality, the active fiber is coiled with a minimum diameter of 10 cm, and water-cooled on a heat sink. The output beam is collimated by a collimator (CO), then the power meter measures the output power, and the M^2^ tester measures M^2^, as shown in Fig. 1.

## Results and Discussion

**Figure 2:**
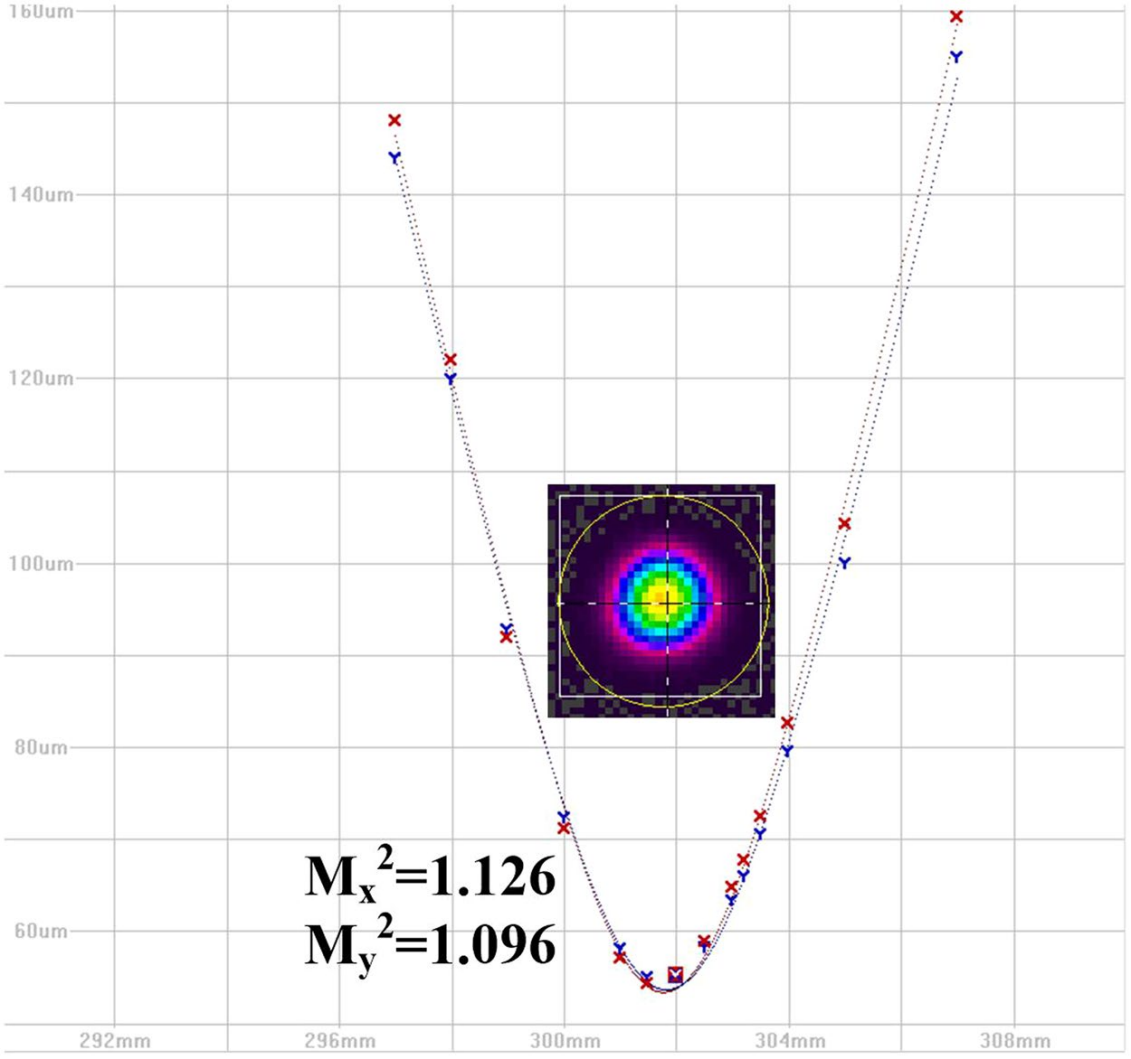
The beam quality of seed laser I.

First, the mode degradation characteristics for the high beam quality system has been studied, and the beam quality factor M^2^ measured by 4-sigma method is employed to feature the beam quality of the system. The fusion splice points of the seed were carefully handled to minimize the excitation of HOM, and the beam quality factor M^2^ of seed laser after the main amplifier is measured three times, and the measured results are ${{\rm{M}}}_{x}^{2}$ = 1.126, ${{\rm{M}}}_{y}^{2}$ = 1.096; ${{\rm{M}}}_{x}^{2}$ = 1.110, ${{\rm{M}}}_{y}^{2}$ = 1.080; ${{\rm{M}}}_{x}^{2}$ = 1.121, ${{\rm{M}}}_{y}^{2}$ = 1.079, one of the measured results is shown in Fig. 2. The output power of seed laser is 100 W. This seed is marked as seed I. The M^2^ is calculated by $\sqrt{({M}_{x}^{2}+{M}_{y}^{2})/2}$, and the average value of M^2^ for seed I is 1.102.

**Figure 3:**
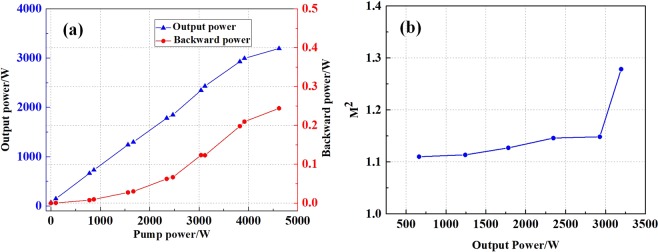
(**a**) The output power and backward power versus pump power for laser, and (**b**) the M^2^ versus output power of laser with seed I.

Injecting the seed I into the main amplifier, the output power and backward power versus pump power are shown in Fig. 3(a). One can see that the output power increases linearly with pump power firstly. When the pump power is 3920 W, the output power is 2995 W, and the optical-to-optical efficiency is 74.5%. However, when the output power exceeds 2995 W, the optical-to-optical efficiency falls dramatically to 28.8%. One can see that the increase trend of backward power indicates no stimulated Brillouin scattering (SBS) effect, so the power rolling over is the sign of the onset of mode instability^3,6^. Then the beam quality at different output powers is illustrated in Fig. 3(b). When the output power is lower than 3000 W, the beam quality maintains near-diffraction limited. However, when the output power exceeds 3000 W, the beam quality degrades suddenly, and the beam quality is ${{\rm{M}}}_{x}^{2}$ = 1.282, ${{\rm{M}}}_{y}^{2}$ = 1.275 at 3196 W, which further confirms the onset of mode instability and the threshold of mode instability is about 3 kW.

**Figure 4:**
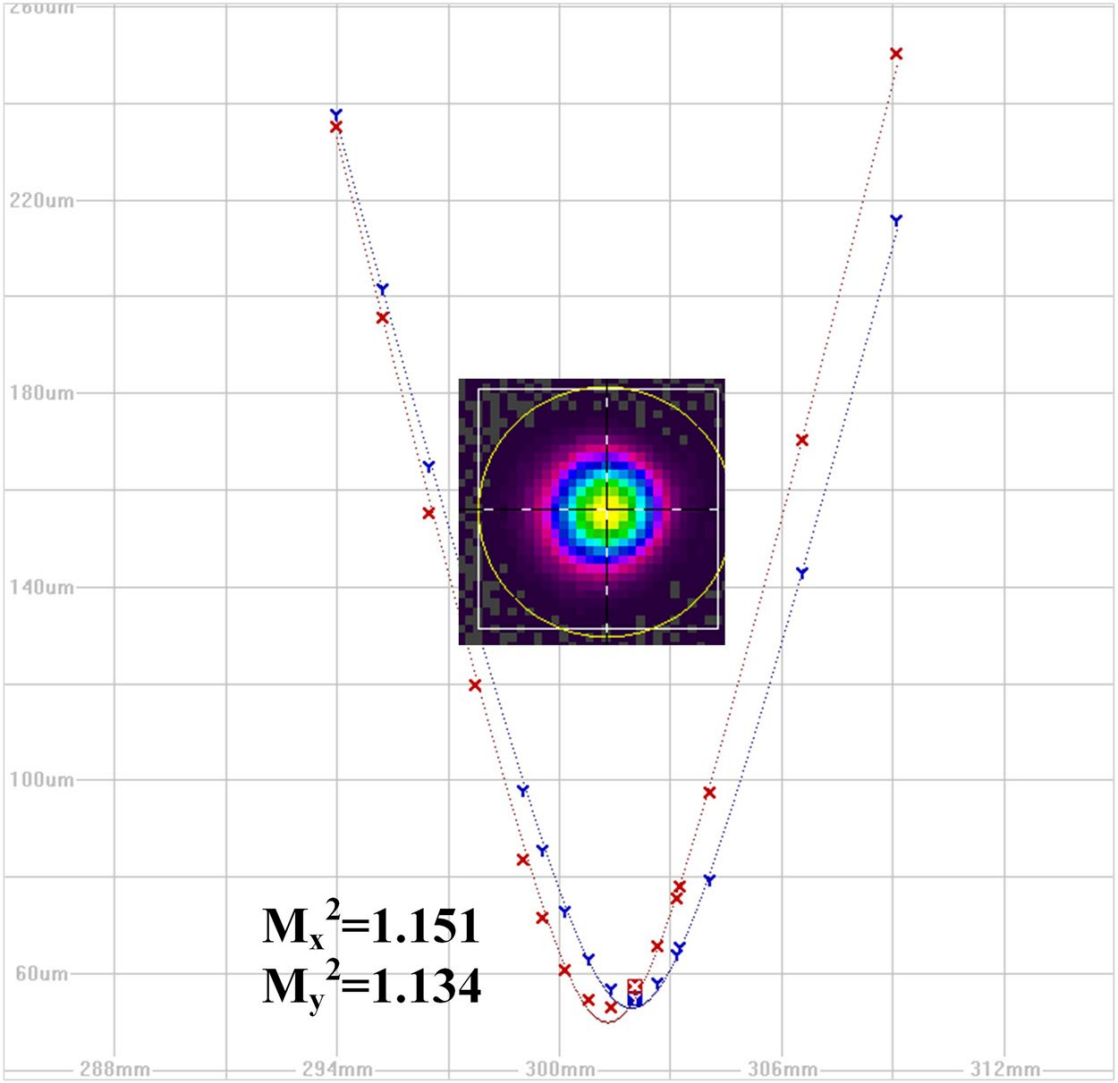
The beam quality of seed laser II.

Then, we intentionally offset spliced the fusion point between the first preamplifier and the second preamplifier (point 1 in Fig. 1) in the seed laser to excite HOM and deteriorate the beam quality of seed laser. This seed is marked as seed II. The standard 10/125 fiber with 0.08 numerical aperture is single mode, and the HOM can not propagate in this fiber. However, further investigation found that because the fiber length in a fiber amplifier is only a few meters, higher-order modes can still propagate in a single-mode fiber amplifier^27^. In addition, the refractive index profile of the practical fiber is not perfect step index^28^, which may lead to that HOM can propagate in the fiber. The output power of seed II is also 100 W. The beam quality of the seed II after the main amplifier is also measured three times, and the results are ${{\rm{M}}}_{x}^{2}$ = 1.151, ${{\rm{M}}}_{y}^{2}$ = 1.134; ${{\rm{M}}}_{x}^{2}$ = 1.158, ${{\rm{M}}}_{y}^{2}$ = 1.130; ${{\rm{M}}}_{x}^{2}$ = 1.148, ${{\rm{M}}}_{y}^{2}$ = 1.140. One of the measured results is shown in Fig. 4, and the average value of M^2^ for seed II is 1.144. The M^2^ of both seed I and seed II are measured three times, so the difference of beam quality is not caused by measurement errors. One can see that the degradation of the beam quality after the seed laser passed the main amplifier is not obvious. This is due to that the fiber is coiled in small size, which induces high bend loss for HOM and the degradation of whole system beam quality without the pump power is not obvious.

**Figure 5:**
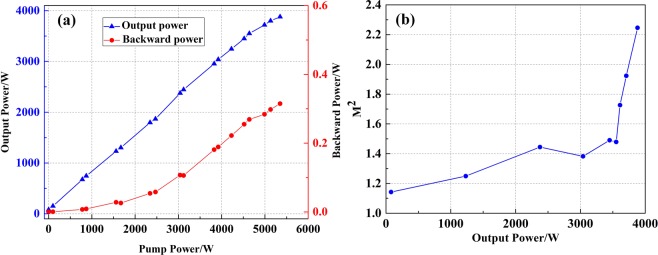
(**a**) The output power and backward power versus pump power for laser, and (**b**) the M^2^ versus output power of laser with seed II.

When the seed II is injected into the main amplifier, the output power of amplifier increases with the scaling of pump power. The backward power is monitored by the port 1of ISO. Figure 5(a) shows output power and backward power versus pump power. At first, the output power increases linearly with pump power. When the pump power is 4640 W, the output power is 3550 W, and the optical-to-optical efficiency is 75.0%. However, when the output power exceeds 3550 W, the output power rolls over, and the optical-to-optical efficiency begins to fall. For main amplifier, the measured M^2^ versus output power is illustrated in Fig. 5(b). With the scaling of output power, the beam quality degrades continuously. At first, the beam quality degrades slowly, and the beam quality is ${{\rm{M}}}_{x}^{2}$ = 1.486, ${{\rm{M}}}_{y}^{2}$ = 1.472 at 3550 W. This confirms that the HOM is excited and the amplification gain of HOM is higher, which gradually surpasses the bend loss and results to the increase of HOM fraction. When the output power exceeds 3550 W, the beam quality experienced sudden degradation to ${{\rm{M}}}_{x}^{2}$ = 2.265, ${{\rm{M}}}_{y}^{2}$ = 2.227 at 3880 W, which indicated the onset of dynamic mode instability, and the dynamic MI threshold is about 3550 W. One can see that mode instability threshold is higher for low beam quality case.

**Figure 6:**
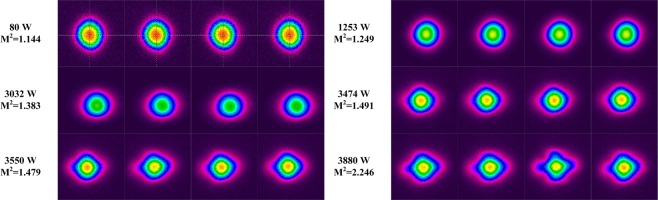
The beam profile at several output powers.

To further understand the power behavior, the beam profiles are monitored by CCD camera at 32 Hz frame frequency, and Fig. 6 shows the consecutively sampled beam profiles at several output powers. All images are displayed with their corresponding power. It is known that the mode instability take place in the kHz-regime, and using an ordinary CCD yields hence strong averaging. The CCD can still capture the dynamical spatial character of mode instability^24,29,30^. It reveals that the output beam distorted when the output power increases from 80 W to 3550 W, and the beam spot evolves from near circular beam into an elliptical ones, which is due to the continuous increase of HOM fraction as the gain of HOM outweighs the bend loss of HOM. One can also see that the beam spot at different time is nearly the same, which means that the degradation is static and the increase of HOM is due to the amplification of Yb gain instead of the nonlinear gain of mode instability. Then a more obviously distorted beam profile is observed when the output power exceeds 3550 W, and the beam spots at consecutively sampling time are quite different, which means that beam profile fluctuates dynamically and the mode instability has set in.

**Figure 7:**
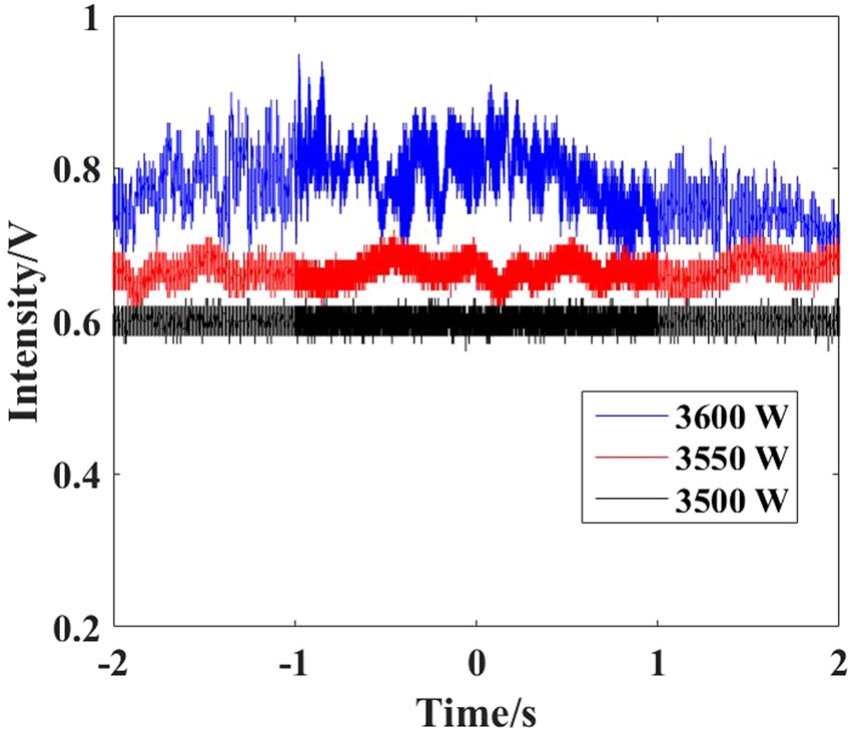
The time traces at different output powers.

To confirm the MI threshold, the time-domain traces at different output power are measured and added by employing the method in^31,32^, as shown in Fig. 7. One can see the output power remains stable at 3500 W, and the output power begins to fluctuate at 3550 W, and fluctuate more obviously with the scaling of output power. The time traces further proved that the dynamic MI threshold is about 3550 W.

Combining the output efficiency, shown in Fig. 5(a), it can be concluded that the static mode coupling will not change the output efficiency, which means that the HOMs caused by static mode degradation are still supported by the fiber system. However, the dynamic mode coupling or mode instability will make the optical-to-optical efficiency fall quickly, and it is observed that the output power will fluctuate in the experiment when dynamic beam degradation occurs. Moreover, the M^2^ will increase quickly when the dynamic beam degradation occurs comparing to static mode degradation, as shown in Fig. 5(b). This is due to that the onset of dynamic mode instability introduces new and more HOMs^33^, which will be leaked into the fiber cladding due to bend loss, and cannot be supported in the bend fiber^3,29^. Due to that the laser linewidth is dozens of GHz, the mode walk-off time propagating through the gain fiber length L was larger than the signal coherence time^34,35^, so the excited HOM, which origins from the HOM content induced by inferior splicing point^36^, can be assumed to superpose incoherently with the FM. Due to the incoherent superposition, the superposed intensity is symmetric, which means that the incoherent HOM has slight effect on the mode instability^7,37^. However, due to that the gain of HOM is larger^38,39^, the presence of incoherent HOM leads to static beam coupling as the power increases, which agrees with the experimental observation.

In the fiber amplifier, the dynamic mode degradation is inherent, and it will occur when the output power exceeds MI threshold. The static mode degradation is caused by the incoherent superposition of FM and HOM in seed due to the limited coherent length, which will prohibit dynamic mode energy coupling but enable static mode degradation for HOM experiences larger gain in the amplifier^38,39^. For the seed II, the offset spliced fusion point will introduce incoherent HOM, which is scaled in amplifier, and lead to static mode degradation. In addition, due to that the dynamic mode coupling is caused by the power coupling between FM and coherent HOM, and the incoherent HOM is included in total output power, so the presence of incoherent HOM will lead to the fact that the measured MI threshold is higher. For the laser with deteriorate beam quality seed, the incoherent HOM content is more than the laser with good beam quality seed, so the measured dynamic MI threshold is higher. This agrees with the phenomena reported in^40^, where the mode instability threshold increases as the bend diameter increases and the fraction of HOM increases. This is not in contract to the findings in^24^, where the seed operates in single frequency states and HOM is superposed with FM coherently. The increase of coherent HOM fraction results in lower MI threshold power^13^.

Comparing the experiment results of these two seed lasers, one can conclude that the mode degradation characteristics of high power fiber amplifier are related to the beam quality or mode excitation of the system. As for the seed with high beam quality, there is nearly no static mode degradation with the scaling of output power, and the degradation of beam quality is slight. The output efficiency will drop and the output power will fluctuate after the onset of dynamic mode degradation. For the seed with slightly lower beam quality, the mode degradation of laser experiences two different regimes as the power increases, a static one and a dynamic one. The static mode degradation consists of a coupling from FM to HOM and there is no dynamic behavior. In this process, the output efficiency of amplifier will not change, and the beam quality M^2^ degrades. After the dynamic mode degradation sets in, the beam quality degrades sharply, which is different from that observed in the aforementioned case. The threshold due to (dynamic) TMI effect is higher for the laser with deteriorate beam quality seed.

## Conclusion

In conclusion, the mode evolution characteristics of the fiber laser with different beam quality have been studied, which reveals that the beam quality of the seed laser plays important role in determination the mode characteristics of the fiber laser system. The results present that there is only dynamic mode degradation in amplifier when a high beam quality laser was employed to seed the amplifier. For the case with a deteriorate beam quality, the beam quality degrades in static at first as the output power scales, and then the dynamic mode degradation occurs when the output power exceeds a certain threshold, which is higher than that for the same amplifier employing a high beam quality seed laser.
